# Preliminary X-ray CT investigation to link Hounsfield unit measurements with the International System of Units (SI)

**DOI:** 10.1371/journal.pone.0208820

**Published:** 2018-12-20

**Authors:** Zachary H. Levine, Adele P. Peskin, Andrew D. Holmgren, Edward J. Garboczi

**Affiliations:** 1 Quantum Measurement Division, National Institute of Standards and Technology, Gaithersburg, Maryland, United States of America; 2 Software and Systems Division, National Institute of Standards and Technology, Boulder, Colorado, United States of America; 3 Professional Research Experience Program, University of Colorado, Boulder, Colorado, United States of America; 4 Applied Chemicals and Materials Division, National Institute of Standards and Technology, Boulder, Colorado, United States of America; University of Notre Dame, UNITED STATES

## Abstract

**Purpose:**

This paper lays the groundwork for linking Hounsfield unit measurements to the International System of Units (SI), ultimately enabling traceable measurements across X-ray CT (XCT) machines. We do this by characterizing a material basis that may be used in XCT reconstruction giving linear combinations of concentrations of chemical elements (in the SI units of mol/m^3^) which may be observed at each voxel. By implication, linear combinations not in the set are not observable.

**Methods and materials:**

We formulated a model for our material basis with a set of measurements of elemental powders at four tube voltages, 80 kV, 100 kV, 120 kV, and 140 kV, on a medical XCT. The samples included 30 small plastic bottles of powders containing various compounds spanning the atomic numbers up to 20, and a bottle of water and one of air. Using the chemical formulas and measured masses, we formed a matrix giving the number of Hounsfield units per (mole per cubic meter) at each tube voltage for each of 13 chemical elements. We defined a corresponding matrix in units we call molar Hounsfield unit (HU) potency, the difference in HU values that an added mole per cubic meter in a given voxel would add to the measured HU value. We built a matrix of molar potencies for each chemical element and tube voltage and performed a singular value decomposition (SVD) on these to formulate our material basis. We determined that the dimension of this basis is two. We then compared measurements in this material space with theoretical measurements, combining XCOM cross section data with the tungsten anode spectral model using interpolating cubic splines (TASMICS), a one-parameter filter, and a simple detector model, creating a matrix similar to our experimental matrix for the first 20 chemical elements. Finally, we compared the model predictions to Hounsfield unit measurements on three XCT calibration phantoms taken from the literature.

**Results:**

We predict the experimental HU potency values derived from our scans of chemical elements with our theoretical model built from XCOM data. The singular values and singular vectors of the model and powder measurements are in substantial agreement. Application of the Bayesian Information Criterion (BIC) shows that exactly two singular values and singular vectors describe the results over four tube voltages. We give a good account of the HU values from the literature, measured for the calibration phantoms at several tube voltages for several commercial instruments, compared with our theoretical model without introducing additional parameters.

**Conclusions:**

We have developed a two-dimensional material basis that specifies the degree to which individual elements in compounds effect the HU values in XCT images of samples with elements up to atomic number *Z* = 20. We show that two dimensions is sufficient given the contrast and noise in our experiment. The linear combination of concentrations of elements that can be observed using a medical XCT have been characterized, providing a material basis for use in dual-energy reconstruction. This approach provides groundwork for improved reconstruction and for the link of Hounsfield units to the SI.

## Introduction

The Hounsfield Unit has enjoyed widespread application in the field of XCT. Nevertheless, from the point of view of a metrological institute, its definition is somewhat incomplete. The definition is

$$\begin{array}{c} 1000\frac{\mu -\mu_{\text{water}}}{\mu_{\text{water}}-\mu_{\text{air}}}. \end{array}$$ (1)

where *μ* is the x-ray attenuation parameter. However, *μ* varies with photon energy, so precisely which x-ray energy *μ* is to be found is left unspecified. Commercial x-ray tomography machines are based on x-ray tubes with a broad spectrum. The x-ray spectrum can vary even within one machine by adjusting the tube voltage. This can be done intentionally to introduce material contrast through “dual energy” scans, i.e., using two values for the x-ray tube voltage. The variation of the HU values in the image as a function of tube voltage implies minimally that the definition of the Hounsfield unit needs to be augmented by the tube voltage.

Multi-energy acquisition has been used to overcome this problem with the Hounsfield unit. Two measurements at different voltages will produce two different sets of resulting Hounsfield units. Because attenuation coefficients of different materials vary differently across voltage ranges, this provides more information to separate materials. Many examples of multi-energy acquisition have been discussed in the literature to aid in medical and biological imaging [1–4] and to avoid beam hardening. [5] A review of the area has appeared recently. [6] Reconstructions are typically performed for each tube voltage independently and then combined after the fact.

The x-ray attenuation parameter *μ*, as tabulated in XCOM [7] is intended to be applied to single photon energies. In such a case, the x-ray intensity follows Beer’s law of attenuation. In passing through material with thickness *x*, the intensity follows

$$\begin{array}{c} I\left(x\right)=I_{0}e^{-\mu x}. \end{array}$$ (2)

Due to the spectrum at any particular voltage, the attenuation will not be exponential and some other rule will be followed. The definition of the attenuation has been refined to be an integral over the applied energies to reflect this. Different spectra attenuate at different rates. If several materials, indexed by *i*, are present, there are multiple *μ*_*i*_ which need to be summed over. The situation is somewhat complicated and the detector-weighted spectral average of the attenuation coefficient normalized to that of a reference material is given by [8–10]

$$\begin{array}{c} \mu^{*}=\frac{\int \;dE\;S(E)\mu (E)}{\int \;dE\;S\left(E\right)\mu^{\left(0\right)}\left(E\right)}, \end{array}$$ (3)

where *E* is the x-ray photon energy, *S*(*E*) is the product of the spectrum *I*(*E*) times the single-photon signal strength of the detector *D*(*E*), and *μ* is the attenuation coefficient (in m^−1^), and *μ*^(0)^ is the attenuation coefficient of the reference material, namely water. Indices for the tube voltage, which determine *S*(*E*), and the material, which determines *μ*, are suppressed.

In metrology, our first question is “What is the measurand?”, i.e., what is being measured? We would like to be able to relate the output in Hounsfield units to its contributions from each material component at each energy level used for an image. As a metrological institute, we prefer that measurements be linked to SI units. We need to carefully define what is being measured, how those measurements relate to x-ray tube voltages, and the dimensionality of what we are measuring. We are trying to determine the quantity of some substance in a volume, so the relevant units are the mole or kilogram (for the quantity of material) and the meter (to make the volume it is held in). If many materials are present, we need to further refine our measurand to include types of moles or kilograms (e.g., moles of carbon atoms vs. moles of oxygen atoms). Broad spectrum x-ray attenuation is fairly insensitive to chemical bonds and just depends on the chemical elements present. This is known as the independent-atom approximation [11] and, in particular, the XCOM tables make this assumption. At its base, the concept of dimensionality is this: if imaging at two different voltages supplies additional information to separate materials in an image, does taking measurements with a third tube voltage supply additional information?

Various authors have concluded the intrinsic dimensionality of the XCT measurement is two [2, 12] or four dimensions. [13, 14] Alvarez [15] noted that the dimensionality depends on the signal-to-noise ratio and suggested that a third dimension allowing for the discrimination of adipose tissue could be recovered under favorable conditions. Recently, Lalonde and Bouchard [9] suggested that two tube voltages were required for soft tissue and an additional two tube voltages could be used for bony tissue, leading to a suggestion that measurements at three or four tube voltages would have an advanges over dual energy XCT for natural (i.e., not contrast enhanced) tissue. [16] Here, we will differ somewhat from previous studies in looking directly at the the intrinsic dimensionality of the Hounsfield unit measurements as a function of tube voltage instead of considering the intrinsic dimensionality of the x-ray cross sections themselves.

Let us assume that only a subset of the chemical elements might be present in our sample. Here we take *Z* = 1 to *Z* = 20 for definiteness. The most information one could hope to extract from a multiple energy XCT reconstruction is the quantity of each of the twenty chemical elements which is present in each voxel. In practice, the x-ray attenuation properties of the first 20 chemical elements are too similar to distinguish them individually with medical x-rays. Yet there is some material effect. For definiteness, and anticipating our later results, we will take the dimension of the space as two in the discussion going forward.

We can view the XCT measurement as a mapping of our length 20 vector (i.e., the 20 numbers which give the concentration of each chemical element at each voxel) into just two numbers, the HU readings at two tube voltages. By hypothesis, the result of a third tube voltage is predictable from these two values (else we could acquire more information through a third tube voltage). Assuming the system is linear in the concentration, a fact tested earlier [17] and demonstrated in cases here as well, this implies that there are two vectors—two linear combinations of concentrations of chemical elements—which we can measure in XCT. If two substances, such as water and simulated water, project to the same point in the 2D subspace, we cannot distinguish between them in an XCT scan.

The purpose of this paper is to characterize this linear combination of concentrations of chemical elements which are observable in XCT. By defining an HU potency measure, which is built upon the dependence of the Hounsfield unit on tube voltage for a particular material, we can overcome the shortcoming of the current Hounsfield unit to move forward two (long-range) objectives: (1) to provide a link to the SI so that XCT measurements can be traceable and defined by moles, kilograms, and meters which are invariant across XCT machines, and (2) for use in a multi-energy XCT reconstruction algorithm which builds in the necessary physics to avoid artifacts such as cupping. [18]

## Materials and methods

### Theory

**Fig 1:**
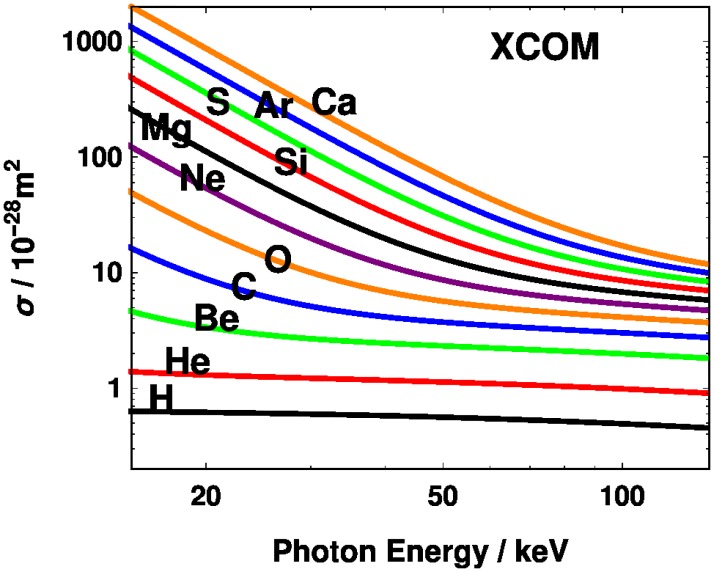
Cross sections for the first 20 chemical elements from XCOM, [7] with curves shown only for hydrogen and even atomic numbers.

Theoretical cross sections for the first 20 elements were obtained from XCOM [7] and are shown in Fig 1. Eq (3) assumes there is no beam hardening, [8] either because it is not present physically in a thin sample or because it is taken into account in a reconstruction algorithm.

To separate out the effect of density from the type of material, we use the decomposition of the attenuation length into the product of an extensive and intensive quantity [19]

$$\begin{array}{c} \mu (E)=n\sigma (E) \end{array}$$ (4)

where *n* is the number density of the particle of interest and *σ* is the cross section. In this context, the particles can be an atom, a molecule, or a formula unit of a polymer or a crystal. Defining the molar potency for the Hounsfield unit to be

$$\begin{array}{c} \eta^{\left(n\right)}=\frac{\int \;dE\;S(E)\sigma (E)}{\int \;dE\;S\left(E\right)\sigma^{\left(0\right)}\left(E\right)}. \end{array}$$ (5)

Eqs (3), (4) and (5) may be combined to yield

$$\begin{array}{c} \eta^{\left(n\right)}=\mu^{*}\frac{n^{\left(0\right)}}{n} \end{array}$$ (6)

where *n* is the number density of the particle of interest and *n*^(0)^ is the number density of water. (The molar density is related to the number density by the Avogadro constant.) We also define a closely related quantity, the mass potency for the Hounsfield unit,

$$\begin{array}{c} \eta^{\left(\rho \right)}=\mu^{*}\frac{\rho^{\left(0\right)}}{\rho }, \end{array}$$ (7)

where the *ρ* is the mass density and *ρ*^(0)^ is the mass density of water. Both *η*^(*n*)^ and *η*^(*ρ*)^ are intensive properties of materials. In contrast, μ* values are extensive properties in the sense that they depend on the density of the material in a given volume.

We used powder samples confined to plastic bottles. Due to packing, a given powder sample had an unknown density and an irregular volume. We did not need to find these because of the following relation. The mass *M* of the powder is given by

$$\begin{array}{c} M=\int \;d\vec{r}\;\rho \left(\vec{r}\right)=\frac{\rho^{\left(0\right)}}{\eta^{\left(\rho \right)}}\int \;d\vec{r}\;\mu^{*}\left(\vec{r}\right). \end{array}$$ (8)

The mass *M* was found experimentally, *ρ*^(0)^ was taken from the literature, [20] and the integrals of $\mu^{*}\left(\vec{r}\right)$ were found from image processing. This is sufficient to determine *η*^(*ρ*)^. If the stoichiometry of the powder is known, *η*^(*n*)^ may be found as well, assuming the isotopes are present in their natural abundance.

**Fig 2:**
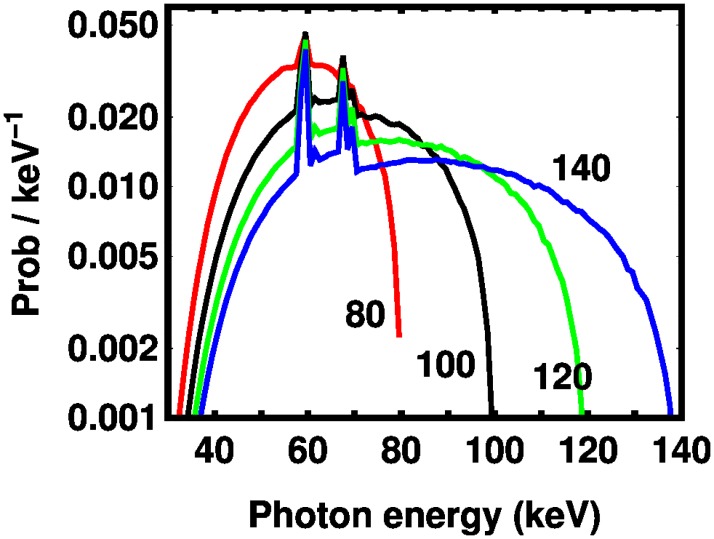
Spectra for tube voltages of 80 kV, 100 kV, 120 kV, and 140 kV calculated from TASMICS, [21] multiplied by a linear ramp function to represent the detector response, assuming a filter of 23.3 mm Al, which was obtained by a fit (see text).

Our estimate of the spectrum was given by the tungsten anode spectral model using interpolating cubic splines (TASMICS). [21] We chose an aluminum filter, a material used in x-ray standards work, [22] and used the thickness as a parameter in a least squares fit to the experimental XCT data. We assumed that the single-photon signal strength is linearly proportional to the photon energy for our model of *D*(*E*). [23] The resulting energy-weighted spectra are given in Fig 2 and show a substantial cut-off below 30 keV. We postpone discussion of how the experimental data was acquired to the experimental section. A best fit thickness of $23.3{}_{-4.4}^{+5.6}$ mm in a 95% confidence interval was determined using a *χ*^2^ test based on least squared errors to the logarithm. The logarithm was chosen so that low *Z* elements would not be underweighted. We recognize that our fit does not imply that there is a physical aluminum filter of this thickness. We prefer to think of the present theory as a “physics-based model” because it is not possible to model completely a commercial XCT machine if only because of proprietary information.

To predict the molar HU potency, the value for water of 1.002 AHU kg m^−3^ is taken from the definition of the HU and an assumed density of 998 kg m^−3^. [20] The corresponding value in moles may be found using the molecular formula H_2_O and the molar mass of water, 18.015 g/mol. It is assumed that the x-ray cross section for any compound is the sum of the cross sections for the constituent atoms which is called the independent atom approximation.

### Experiment

We placed 32 samples in nominally identical 15 mL polyethylene bottles (ThermoFisher Scientific, Waltham, MA, USA) including 30 powders, water, and air. The compounds are listed in Table 1. The powders were chosen to represent the chemical elements in the first 20 atomic numbers. To ensure that the effect of any given element was not masked, it was either taken as the elemental form or included as the highest atomic number *Z* in one compound and present in two other compounds. An exception was made for nitrogen because a sufficiently safe powder could not be located.

**Table 1:** The observed mass potency *η*^(*ρ*)^ for the given compounds for each tube voltage. The initials refer to the supplier: AA (Alfa Aesar, Haverhill, MA, USA), SA (Sigma-Aldrich, St. Louis, MO, USA), and TM (NIST Thermodynamic Metrology Laboratory, Gaithersburg, MD, USA).

			80 kV	100 kV	120 kV	140 kV
agar	C_14_H_24_O_9_	SA	0.9906	0.9942	0.9968	1.0000
aluminum	Al	AA	1.5110	1.3570	1.2710	1.2190
ammonium bicarbonate	NH_5_CO_3_	SA	0.9687	0.9752	0.9776	0.9815
ammonium phosphate monobasic	NH_6_PO_4_	SA	1.2760	1.2010	1.1590	1.1330
calcium carbonate	CaCO_3_	AA	2.1560	1.8590	1.6900	1.5870
calcium chloride	CaCl_2_	SA	2.8090	2.3400	2.0700	1.9050
calcium phosphate	Ca_3_P_2_O_8_	SA	2.1910	1.8810	1.7040	1.5940
calcium sulfate dihydrate	CaSO_6_H_4_	SA	1.9970	1.7500	1.6070	1.5220
graphite	C	AA	0.8808	0.9047	0.9230	0.9365
hydroxyapatite	Ca_5_P_3_O_13_H	SA	2.2670	1.9430	1.7590	1.6500
kaolin	Al_2_Si_2_O_9_H_4_	SA	1.4120	1.3090	1.2490	1.2160
magnesium carbonate	MgCO_3_	SA	1.1350	1.1010	1.0910	1.0870
magnesium chloride hexahydrate	MgCl_2_H_12_O_6_	SA	1.6180	1.4610	1.3710	1.3160
magnesium nitrate hexahydrate	MgN_2_H_12_O_12_	SA	1.0300	1.0210	1.0110	1.0100
magnesium oxide	MgO	SA	1.3080	1.2290	1.1890	1.1640
potassium carbonate	K_6_CO_3_	SA	2.1060	1.8120	1.6400	1.5370
potassium chloride	KCl	SA	2.6720	2.2340	1.9820	1.8250
potassium nitrate	KNO_3_	AA	1.8270	1.6010	1.4750	1.3970
potassium phosphate dibasic	K_2_HPO_4_	SA	2.2200	1.9040	1.7250	1.6150
potassium phosphate monobasic	KH_2_PO_4_	SA	1.8050	1.5860	1.4600	1.3830
potassium sulfate	K_2_SO_4_	SA	2.1860	1.8770	1.7030	1.5930
silicon	Si	AA	1.7650	1.5580	1.4440	1.3720
sodium acetate	NaC_2_H_3_O_2_	SA	1.0330	1.0160	1.0070	1.0030
sodium bicarbonate	NaCHO_3_	SA	1.0230	1.0000	0.9875	0.9806
sodium carbonate	NaCO_3_	SA	1.0720	1.0360	1.0170	1.0070
sodium chloride	NaCl	SA	1.9240	1.6660	1.5220	1.4320
sodium phosphate dibasic	Na_2_HPO_4_	SA	1.2770	1.1900	1.1410	1.1120
sulfur	S	AA	2.2460	1.9270	1.7430	1.6290
tricalcium phosphate	Ca_3_P_2_O_8_	SA	2.3720	2.0390	1.8480	1.7320
urea	CH_4_N_2_O	SA	0.9543	0.9657	0.9712	0.9775
water	H_2_O	TM	1.0290	1.0310	1.0320	1.0340

The mass of each powder and the water was determined by weighing each bottle before and after the powder or water was placed in it. The powder masses ranged from 1.896 g (MgCO_3_) to 16.860 g (NaCl). The mass of the water was 14.544 g. The masses of the empty bottles ranged from 5.705 g to 5.753 g with a mean of 5.721 g and a standard deviation of 0.012 g. Because the standard deviation was a small fraction of the powder mass, differences in the bottle masses were neglected when performing the subtraction of an empty bottle described next.

To simplify the problem of segmentation, we included a small region of the exterior of each bottle defined by setting a threshold and subtracted out the integral of an empty bottle. Varying the threshold value over a full range of 40 HU led to variations in the difference of the integrated values typically of 0.2% and at most 0.5%. Reconstruction of the powder may be convolved with the interior edge of the bottle, but since the thickness of the bottle was large compared to the spatial resolution of the algorithm, we were able to obtain accurate values for the integral in Eq (8).

The samples were placed on a low density but rigid foam and imaged at 80 kV, 100 kV, 120 kV, and 140 kV, using a Siemens SOMATOM XCT at the Veterans’ Administration hospital in Baltimore, Maryland, USA. An axial scan was taken using the Syngo CT 2012B chest routine protocol. The DICOM files from the XCT scans are available; see Public Data section below. The samples were placed in the XCT so that no two bottles were imaged in the same slice. Other than the tube voltage, the same parameters were used for all four images, including the dimensions of the voxels (0.3125 mm × 0.3125 mm × 0.625 mm), the integrated tube current (100 mAs), the reconstruction algorithm, and the field of view. The protocol with some additional details is available. [24]

## Results

### Comparison of model to present experiment

**Fig 3:**
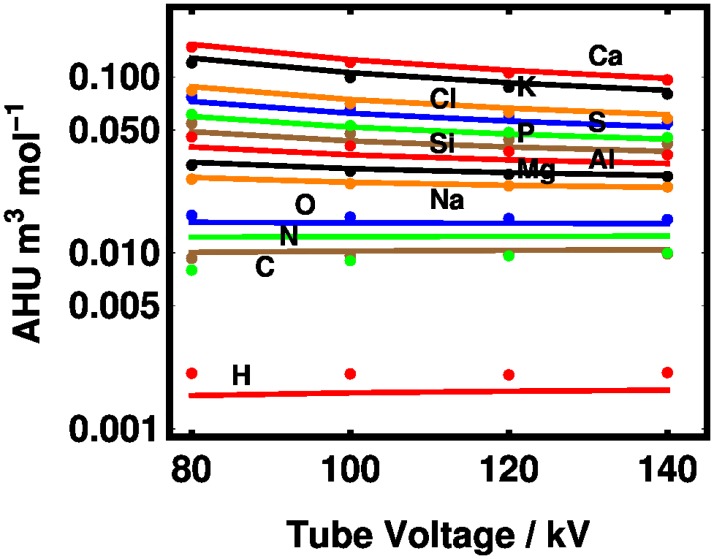
Theoretical prediction of AHU per mole density of a given element according to the XCOM cross sections of Fig 1 (lines) and the best-fit spectrum-detector model shown in Fig 2 (points). The curves represent the 13 elements used in the experiment (H, C, N, O, Na, Mg, Al, Si, P, S, Cl, K, and Ca).

The molar HU potency *η*^(*n*)^ is shown in Fig 3 for both theory and experiment. The experimental points were obtained by projecting the data of Table 1 onto a space spanned by the 13 elements in the study (namely, *Z* = 1, 6-8, 11-17, and 19-20). This projection was done by (a) defining a matrix *H* of molecular molar HU potencies indexed by compound and the tube voltage in that order; (b) constructing a matrix *C* from the chemical formulas indexed by compound and atom in that order, and (c) minimizing the residual of

$$\begin{array}{c} {\left|CA-H\right|}^{2} \end{array}$$ (9)

to determine the matrix *A* of atomic molar HU potencies, indexed by atom and tube voltage in that order. This projection was performed separately for each tube voltage. In this way, we reduce the 128 measurements (from 32 samples at 4 tube voltages) to 52 values (13 elements at 4 tube voltages). By definition, data left out is outside of the independent atom approximation.

As mentioned above, the theoretical curves were found by taking the unfiltered TASMICS spectra and introducing an aluminum filter whose thickness is a fitting parameter. Once we fixed the filter thickness, we compared the theoretical model to the data from the powders and water. The higher the atomic number the higher the potency, except for the nitrogen experimental value which appears below carbon. Given the cross sections shown in Fig 1, it is not a surprise that higher tube voltages lead to less elemental contrast. The tendency of the potency to relax to the value for oxygen (the dominant component of water) at higher tube voltages for both theory and experiment may be seen in Fig 3. Absolute differences between the experiment and theory are given in S1 Table in the Supporting Information.

**Fig 4:**
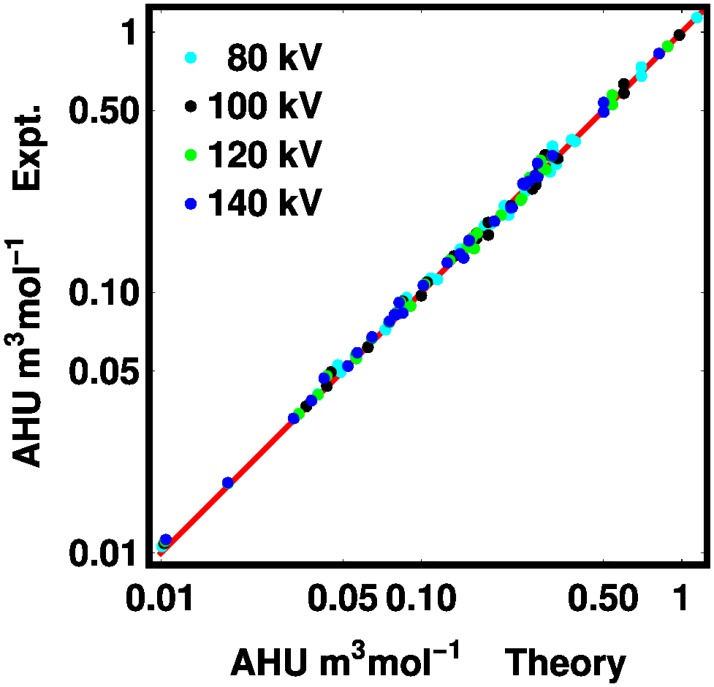
Experimental values for the molar Hounsfield unit potency is shown for both theory and experiment. The red line is the identity function. The colors code the tube voltages as: 80 kV (cyan), 100 kV (black), 120 kV (green), and 140 kV (blue). The plotted points are given in tabular form with the public data.

The comparison of the theory to our experimental powder measurements are shown in Fig 4. The theory gives a good account of the experiment with an root-mean-square (RMS) deviation of 4.8%. We double this figure and round up to say that our theory can predict the HU potency with 10% accuracy with 95% confidence. The absolute errors are listed for each material in S2 Table in the Supporting Information.

### Characterization of practical observables in HU measurements

First, we consider the intrinsic dimensionality of both our theory and our measured data. In practical language, we ask the question, can anything more be learned by performing scans at a second, third, or fourth tube voltage?

A Singular Value Decomposition (SVD) was applied to both the theoretical and the experimental values of the potency given in Table 1. For the theory, *η*^(*n*)^ was found for all elements from *Z* = 1-20 at the four tube voltages. For our experiment, as discussed above, the values of *η*^(*n*)^ were projected on a space of linear combinations of *η*^(*n*)^ for the elements, as presented in Fig 3.

**Fig 5:**
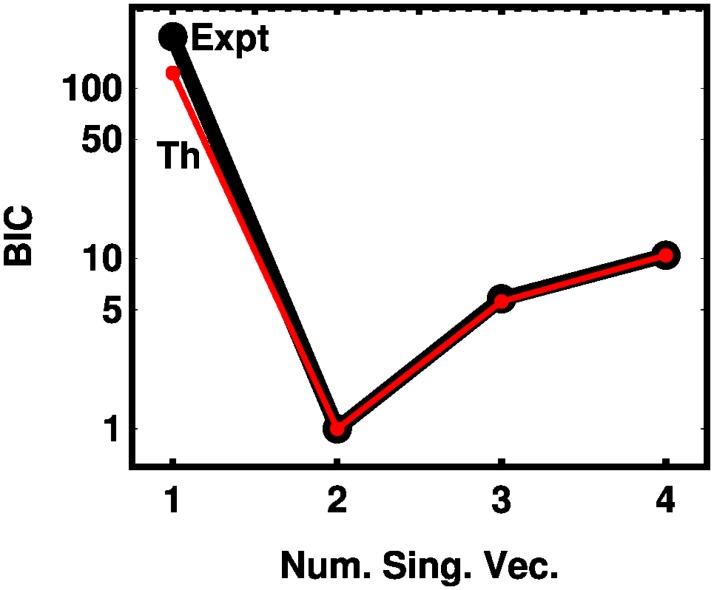
The Bayesian Information Criterion (BIC) according to Eq (10) is shown for the experiment using the sum-of-elements approximation (black) and the model (red). In both cases, the number of parameters in the BIC formula is the the number of singular vectors retained.

The singular values are compared in Table 2. There is semiquantitative agreement for the first two singular values, but for the third and fourth singular values, the theory falls off more sharply. To determine which of these singular values rise above experimental noise, we use the Bayesian information criterion [25]

$$\begin{array}{c} B=2\ln L-N_{p}\ln N_{o} \end{array}$$ (10)

where ln *L* is the log likelihood, *N*_*p*_ is the number of parameters, and *N*_*o*_ is the number of observations. We fit the 128 HU values obtained from the experiment to models that consist of retaining 1, 2, 3, or 4 singular values and vectors. We do this independently for the experimental vectors derived from the independent atom approximation and from the theory. The log likelihood is the *χ*^2^ value obtained from the squared error associated with the four cases. The results are shown in Fig 5. The results show a clear minimum at 2 parameters. The negligible progress in fitting the data with more parameters indicates that the intrinsic dimensionality of the measurement space is two for our experiment. This is true whether our model is taken from experiment using the independent atom approximation or from the theory. It also resolves the issue of the disagreement of the third and fourth singular values: both are consistent with zero in terms of their predictive power. Absolute differences between the experiment and the model are given in S3 Table in the Supporting Information.

**Table 2:** Singular values for the molar HU potency matrix (AHU m^3^ mol^−1^).

Expt.	0.4084	0.0144	0.000347	0.000219
Theory	0.4527	0.0156	0.000020	0.000001

**Fig 6:**
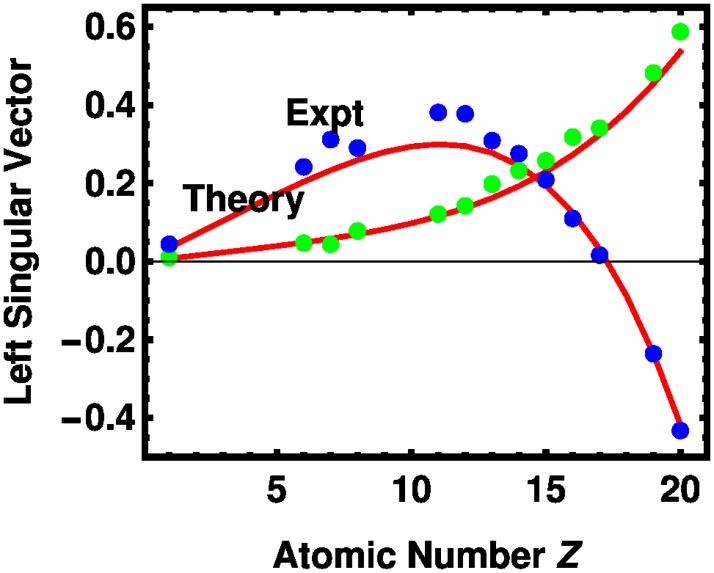
The components of the first two left singular vectors of the molar HU potency matrix for several elements, as indexed by the atomic number *Z*, are shown for theory (red lines) and experiment (first, green dots; second, blue dots).

**Fig 7:**
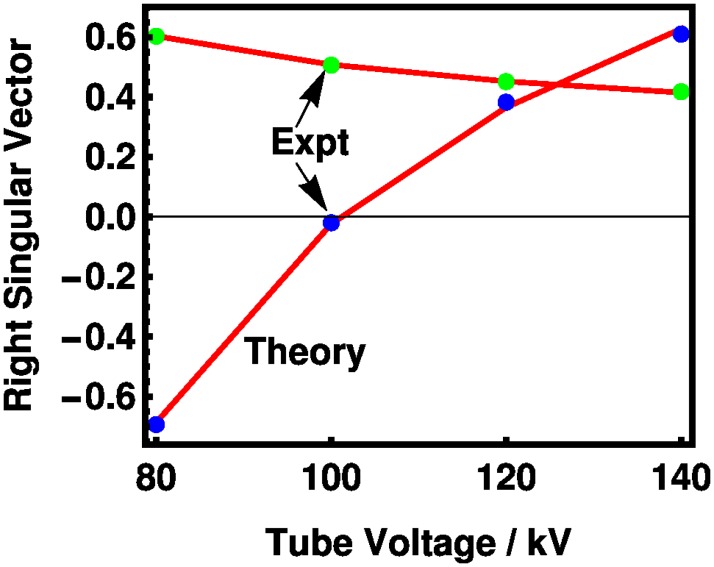
The components of the first two right singular vectors of the molar HU potency matrix, as indexed by the tube voltages, are shown for theory (red lines) and experiment (first, green dots; second, blue dots).

The first two left and first two right singular vectors are presented in Figs 6 and 7, respectively. The experimental and theoretical coefficients track each other fairly well, although nitrogen is out of trend as it was in Fig 3. The first left singular vector, shown in Fig 6, varies approximately as *Z*^2.6^. The left singular vectors are similar to those presented in an early study of tissue attenuation coefficients. [26] For the right singular vectors, the theory tracks the experiment without exception. Absolute differences between the experimental and theoretical values are given in S4 and S5 Tables in the Supporting Information. In our approach, the vector is related to tube voltages, which differs from earlier presentations of the Basis-Vector Model (BVM) [10, 12, 27] in which the independent variable is the photon energy.

### Comparison of model to experiments from the literature

The use of reference materials, or phantoms, to calibrate medical XCT has a long history. Here, we compare our results to several recent measurements of phantoms at multiple tube voltages. In some cases, we compare to results from multi-center trials. Our motivation is to see whether the model developed on one set of compounds for one XCT machine is applicable to a wider set of machines. Such an approach is often referred to in model building as “training” and “validation”.

**Fig 8:**
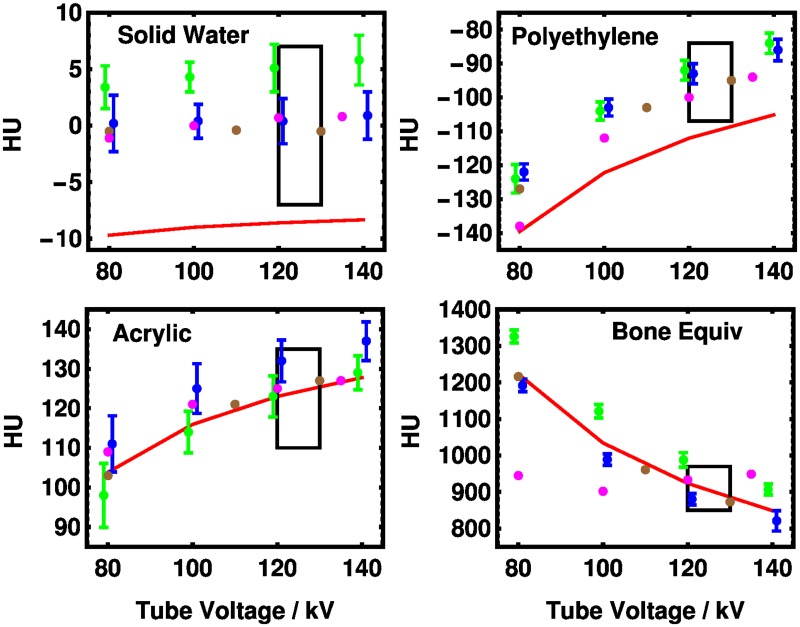
Comparison of model to measurements from the literature. Experimental points are from the measurements of [30] with recommended ranges for 120 kV to 130 kV tube voltages from the American College of Radiology given as black rectangles. The machines are identified in [30] as GE (green), Siemens (blue), Siemens SPECT-CT (brown), and Toshiba (magenta). The data were drawn from 25 GE, 7 Siemens (non-SPECT-CT), 3 Siemens SPECT-CT, and 1 Toshiba scanners. The error bars represent the interquartile ranges reported in the paper for multiple scanners of the same type. Error bars are not given if only a single machine was reported. The values for GE and Siemens have been offset by ∓1 kV, respectively, for clarity. The red line is the present theory using mass fractions and densities as specified by the vendor. [29] “Solid Water” is a water equivalent polymer.

In Fig 8, results from scanning the American College of Radiology (ACR) phantom [28] on 36 XCT machines are shown along with the ranges recommended by the ACR. The model accounts for the data quantitatively with small exceptions for “solid water” and polyethylene. However, in both cases, better agreement would be obtained if the density were 1% higher than given by the manufacturer’s specifications [29] which represents a change of 1 in the most significant figure quoted. A statement of uncertainties of the densities of the reference materials was not available. Differences between our theory and the experimental values are given in S6 Table in the Supporting Information.

**Fig 9:**
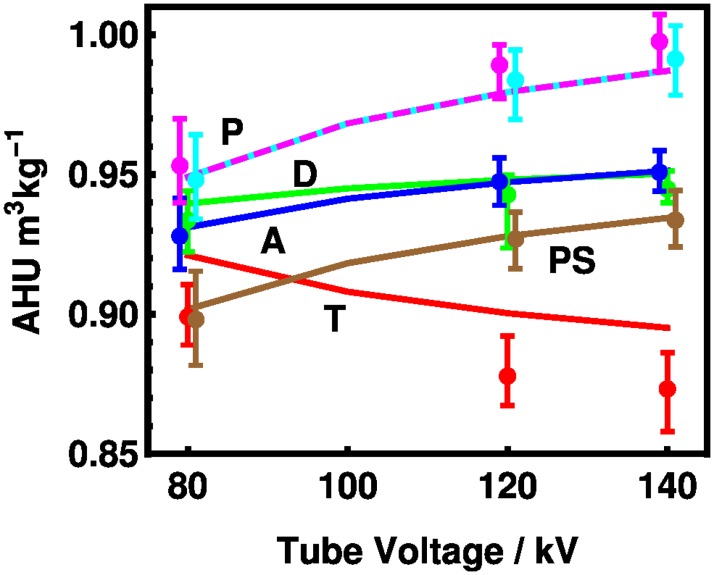
Mass HU potency vs. tube voltage for reference materials from the Catphan phantom. [31] The materials are T (Teflon, or polytetrafluoroethylene C_2_F_4_, red), D (Delrin, CH_2_O, green), A (acrylic, see Fig 8, blue), PS (polystyrene, C_8_H_8_, brown), and P (both low density polyethylene C_2_H_4_ magenta, dashed and polymethylpentene C_6_H_12_ cyan, overwritten) The tube voltages have been offset for clarity by -1 kV for and low density polyethylene and acrylic and by +1 kV for polymethylpentene and polystyrene. Conversions from the reported HU values are scaled by the densities 2160, 1415, 1180, 1040, 925, and 833 kg/m^3^, respectively. The error bars represent the minimum and maximum values as reported by [31] for 8 scanners, scaled by the densities.

In Fig 9, comparison between the model and measurements of the Catphan phantom [31] are given in terms of *η*^(*ρ*)^, the mass Hounsfield unit potency. The model agrees with the experiment within uncertainties in most cases. Teflon, C_2_F_4_, slopes downward in both theory and experiment because its x-ray properties are dominated by an element with atomic number above that of oxygen. The disagreement between theory and experiment could be associated with the density of the Teflon being 2% lower than estimated.

The model, due to its linearity, predicts that polyethylene (C_2_H_4_) and polymethylpentene (C_6_H_12_) have the same potency because they have the same ratio of carbon to hydrogen, and this is observed experimentally. The linearity of Hounsfield units measurements for a system with the same material at different densities, leading to a single value for the mass Hounsfield unit potency, was reported for a series of commercial polyurethane foam samples. [17] Absolute error measurements are given in S7 Table in the Supporting Information.

**Fig 10:**
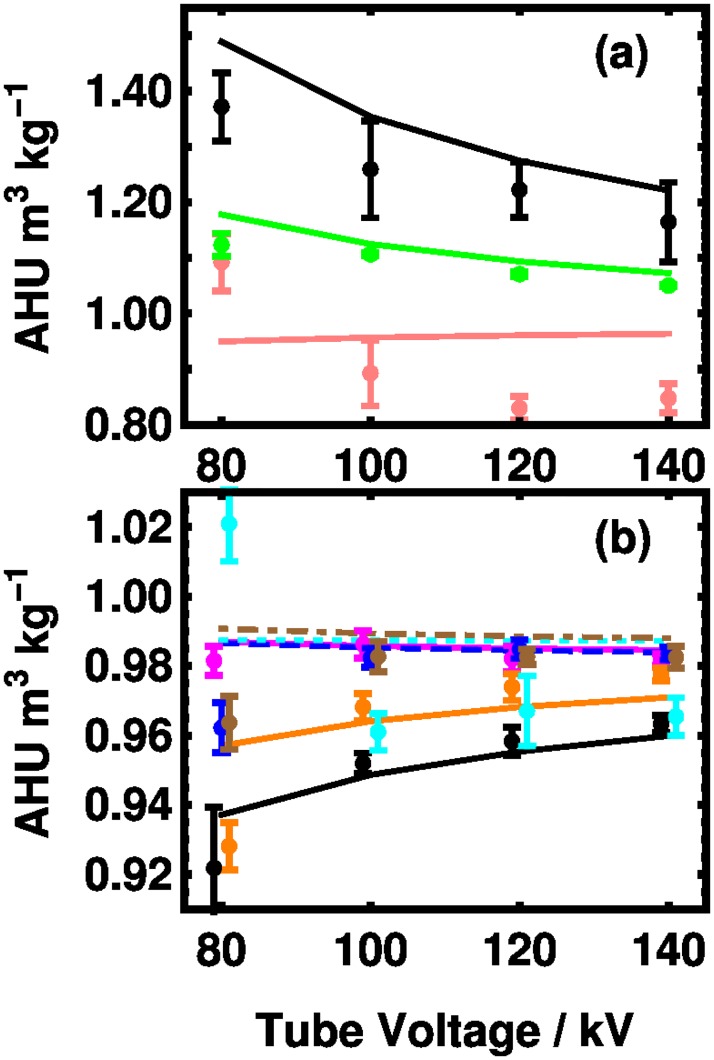
Mass HU potency vs. tube voltage for reference materials from the CIRS Model 62 phantom. [32] The tissue-equivalent materials are: (a) dense bone (black), trabecular bone (green), lung exhaled (salmon) (b) liver (magenta), muscle (blue, dashed), breast (orange), adipose (black) lung inhaled (cyan, dotted), and plastic water (brown, dot dashed). Hounsfield unit data and uncertainties from Ref. [32] have been scaled by the densities. [20] Some data points in (b) have been offset by ±1 kV for clarity.

Finally, data from a single laboratory’s measurement of the CIRS Model 62 phantom, designed for electron density calibration, is shown in Fig 10. The bone-like material given in Fig 10a shows a decreasing potency with tube voltage in both experiment and theory. There is a small disagreement with the magnitude, but not larger than the 10% theoretical uncertainty estimated above. There is a fall off in the potency experimentally for both the inhaled and exhaled lung materials that is not captured by the theory. For the other materials, agreement with the theory is good. Absolute errors between the experimental and theoretical numbers are given in S8 Table in the Supporting Information.

To summarize this section, the theory is compared with several results from the literature where Hounsfield unit values are given for phantom materials at several tube voltages. The theory accounts for the major trend that the mass HU potency rises with increasing tube voltage for materials with low atomic number and falls for those with high atomic number. The results are given quantitatively within experimental uncertainties in many cases, and within the estimated 10% estimated theoretical uncertainty in all cases. Although there is one parameter in the model—the filter thickness—this parameter was not modified after being fit based on our original experimental data.

## Discussion

While dual energy XCT is now common in practice, the reconstructions typically are done for each tube voltage independently and combined after the fact. This work may enable an alternative strategy, determining two materials at each voxel during the reconstruction. [33] We provide a principled way to determine abstract materials for use as a basis in such a reconstruction. The left singular vectors given above characterize how to transform between the two abstract materials and real materials. Any two materials which project to the same point in the two dimensional space spanned by the left singular vectors will be indistinguishable in medical XCT. For example, “solid water” (as used in the ACR phantom) or “plastic water” (as used in the CIRS phantom) is not water, but appears like it in x-ray XCT measurements.

We are able to account quantitatively for a wide range of data obtained on many different XCT machines within theoretical and experimental uncertainties. The best predictions are for substances with a density near water which are dominated by first row elements. Cases with very low density or higher atomic numbers are less well accounted for. The work that we present here should be extended over a larger range of elements. Our smaller study of only elements *Z* ≤ 20 demonstrates its usefulness.

The data in Fig 8 show the differences in HU values for scans across four different XCT machines. In the lower right panel, where HU values for the bone equivalent substance are presented, the range of reported values for the GE systems and for the Siemens systems taken separately is much smaller than the difference between them. For a given tube voltage, there is a vendor-specific difference in the reported values which is not captured by the commonly used notation HU_80_, …, HU_140_. In metrology, we distinguish between precision, the ability to reproduce a measurement under a given condition, with accuracy, the ability for that measurement to be meaningful by some external definition. This is *not* to say that one vendor is right or wrong. Rather, it underscores the need for a more complete definition of the Hounsfield unit, to increase the harmonization of the meaning of the measurements. Such an improved definition must be based on the underlying physics. Our material basis is a first step in enabling a definition which could allow the accuracy of measurement to match the precision which is already established in the field.

As a metrology institute, we are interested in the question of what an XCT can measure in principle. The left singular vectors presented in Fig 6 provide a preliminary answer in the sense that certain linear combinations of quantities of given elements yield equivalent Hounsfield unit values. Fully quantifying such a relationship in a metrologically traceable manner remains a long-term goal which may require co-ordination between vendors and standards organizations to achieve. It may also lead to tighter specifications for reference phantoms which could make XCT measurements more transferable between vendors. In particular, the results for the “Bone Equivalent” material shown in Fig 8 suggests an area where additional standards could lead to harmonization of results among vendors.

The left singular vectors predicted by the model may be a superior basis for reconstruction in that they may minimize crosstalk [34] between material basis vector coefficients in the reconstruction. Use of the left singular vectors also provides a principled way to choose a single material basis vector for a gray scale reconstruction, then refine the result by including an orthogonal vector (the second left singular vector) which is known to have less effect on the reconstruction. Such vectors could be used as a basis in polychromatic algorithms which have been shown to reduce reconstruction artifacts. [5, 35, 36]

In this study, we have emphasized the range of elements that are found in large quantities in the human body and in common XCT phantoms. Dual energy XCT is often used with contrast agents which include elements such as iodine and barium, although non-contrast scans are current practice as well. [16] Elements with K edges in the range of medical x-rays would likely require one or more additional tube voltages, or a scan with a limited spectral range, to provide a complete picture of the patient or sample.

### Extensions of the present work

To extend our results to elements such as iodine and barium, it may be necessary to revisit the XCOM tables themselves. Chantler [37] discussed the uncertainties of the tabulations of x-ray cross sections. He found that tabulated atomic cross sections are accurate to about 2% for cross sections for photon energies at least 20% above the highest K edge in the system. This is true for our data, given that our highest K edge, that of calcium at 4 keV, is well below the vast majority of the spectral intensity. The independent atom approximation is much less reliable near the K edge of a material where the effects of neighboring atoms in x-ray absorption fine structure may be large. [38] Thus our model may not be directly applicable to high *Z* materials such as barium (with its K edge at 37 keV) that are used as XCT contrast agents. A more simple extension of the present work would be to elements of medical interest with K edges below about 30 keV, such as iron. [39, 40]

In this work, we consider conventional multi-energy XCT where the tube voltage is varied with a single energy-integrating detector. In the past few years, photon-energy-resolving detectors have received more attention as they become commercially available. [6, 34, 41] The multi-energy XCT show promise for providing additional material-dependent resolution, particularly if the energy thresholds are co-incident with x-ray K-edges of contrast agents such as gadolinium. The methods used herein could be used to probe the intrinsic dimensionality of material contrast in such systems.

## Conclusions

We performed XCT measurements on 32 materials at four tube voltages on a commercial XCT. We showed that the powder measurements were related to an intensive quantity which we name the mass or molar Hounsfield unit potency. We show how to find this quantity using the measured mass of powder in a given container even though the density of the powder may vary in the container.

We developed a theoretical model for this quantity using tabulated x-ray cross sections, [7] recently available tube spectra, [21] and a measurement model for XCT. [8–10] We introduced one parameter, an Al filter thickness, to describe the filtration of the spectrum which was fit to our measurements. We gave a good account of our data, with the potency given to 10% within a 95% confidence interval. Without changing the fitting parameter, we accounted for measurements of calibration phantoms from the literature done including dozens of medical XCTs on more than ten materials within joint experimental and theoretical uncertainties.

We have established a method to define an orthogonal material basis to transform between abstract and real materials which we hope will be useful in reducing crosstalk between material basis components in tomographic reconstruction algorithms. Our theory is derived from quantities in SI units of moles or kilograms and meters. We hope that the work presented here will play a role in bringing about an SI traceable definition of XCT measurements which are now only reported in Hounsfield units.

## Supporting information

S1 Table Element specific absolute errors of the data from Fig 3. Errors are defined as: (AHU experimental measurement minus AHU theoretical measurement) in units of m^3^ mol^−1^.
(TEX)

S2 Table Material specific absolute errors of the data from Fig 4. Errors are defined as: (AHU experimental measurement minus AHU theoretical measurement) in units of m^3^ mol^−1^.
(TEX)

S3 Table Absolute differences in the Bayesian information criteria (BIC) between theoretical and experimental values from the data of Fig 5. (TEX)

S4 Table Absolute differences between experimental and theoretical values for the first left singular vector of the molar HU potency matrix from Fig 6, followed by values for the second left singular vector. Components are indexed by the atomic number *Z*.
(TEX)

S5 Table Absolute differences between experimental and theoretical values for the first right singular vector of the molar HU potency matrix from Fig 7, followed by values for the second right singular vector at 80, 100, 120, and 140 kV. (TEX)

S6 Table Absolute errors of the data from Fig 8. The experimental measurements in Hounsfield Units (HU) were obtained on CT machines from 4 different CT manufacturers. Measurements at 80, 100, 120, and 140 kV are reported where available. Error is given as the difference between experiment and theory.
(TEX)

S7 Table Absolute errors for reference materials from the Catphan phantom data of Fig 9. The experimental measurements given in AHU m^3^ kg^−1^ taken at 80, 120, and 140 kV are compared with our theoretical predictions. For each of the six materials, experimental values of HU, theoretical values, and the difference between these two quantities.
(TEX)

S8 Table Absolute errors for reference materials from the data of Fig 10. The experimental measurements taken at 80, 120, and 140 kV are compared with our theoretical predictions. For each of the nine materials, experimental values of AHU m^3^ kg^−1^, theoretical values, and the difference between these two quantities.
(TEX)

## References

[1] Phelps ME, Hoffman EJ, Ter-Pogossian MM. Attenuation coefficients of various body tissues, fluids, and lesions at photon energies of 18 to 136 keV. Radiology. 1975;117:573–583. 10.1148/117.3.573 810827

[2] Alvarez RE, Macovski A. Energy-selective reconstructions in x-ray computerized tomography. Phys Med Biol. 1976;21:733–744. 10.1088/0031-9155/21/5/002 967922

[3] Heismann BJ, Leppert J, Stierstorfer K. Density and atomic number measurements with spectral x-ray attenuation method. J Appl Phys. 2003;94:2073–2079. 10.1063/1.1586963

[4] Martinez LC, Calzado A, Rodriguez C, Gilarrantz R, Manzanas MJ. A parametrization of the CT number of a substance and its use for stoichiometric calibration. Phys Medica. 2012;28:33–42. 10.1016/j.ejmp.2011.02.001 21419682

[5] Yu L, Leng S, McCollough CH. Dual-energy CT-based monochromatic imaging. Am J Roentgenology. 2012;117:S9–S15. 10.2214/AJR.12.9121 23097173

[6] McCollough CH, Leng SA, Yu LF, and Fletcher JG, Dual- and multi-energy CT: Principles, technical approaches, and clinical applications Radiol. 2015;276:637–653 10.1148/radiol.2015142631 PMC4557396 26302388

[7] Berger MJ, Hubbell JH, Seltzer SM, Chang J, Coursey JS, Sukumar R, et al. XCOM: photon cross sections database. http://wwwnistgov/pml/data/xcom/indexcfm, retrieved Oct 5, 2016. 1998; p. retrieved Oct. 5, 2016.

[8] Judy PF, Adler GJ. Comparison of equivalent photon energy calibration methods in computed tomography. Med Phys. 1980;7:685–691. 10.1118/1.594722 7464712

[9] Lalonde A, Bouchard H. A general method to derive tissue parameters for Monte Carlo dose calculation with Multi-energy CT. Phys Med Biol. 2016;61:8044–8069. 10.1088/0031-9155/61/22/8044 27779137

[10] Han D, Siebers JV, Williamson JF. A linear, separable two-parameter model for dual energy CT imaging of proton stopping power computation. Med Phys. 2016;43:600–612. 10.1118/1.4939082 26745952 PMC4706548

[11] Poludniowski G, Evans PM, Webb S. Rayleigh scatter in kilovolt x-ray imaging: Is the independent atom approximation good enough? Phys Med Biol. 2009;54:6931–6942. 10.1088/0031-9155/54/22/012 19887715

[12] Williamson JF, Li S, Devic S, Whiting BR, Lerma FA. On two-parameter models of photon cross sections: Application to dual-energy CT imaging. Med Phys. 2006;33:4115–4129. 10.1118/1.2349688 17153391

[13] Midgeley SM. Materials analysis using x ray linear attenuation coefficient measurements at four photon energies. Phys Med Biol. 2005;50:4139–4156. 10.1088/0031-9155/50/17/016 16177536

[14] Bornefalk H. XCOM intrinsic dimensionality for low-Z elements at diagnostic energies. Med Phys. 2012;39:654–657. 10.1118/1.3675399 22320774

[15] Alvarez RE. Dimensionality and noise in energy selective x-ray imaging. Med Phys. 2013;40:111909 10.1118/1.4824057 24320442 PMC3808483

[16] Chi BH, Chang IH, Lee DH, Park SB, Kim KD, Moon YT, et al Low-dose unenhanced computed tomography with iterative reconstruction for diagnosis of ureter stones. 2018;59:389–396. 10.3349/ymj.2018.59.3.389 PMC5889991 29611401

[17] Levine ZH, Li M, Reeves AP, Yankelevitz DF, Chen JJ, Siegel EL, et al A low-cost density reference phantom for computed tomography. Med Phys. 2009;36:286–288. 10.1118/1.3049596 19291968 PMC2673671

[18] Taguchi K and Iwanczyk JS Beam hardening artefacts in computed tomography with photon counting, charge integrating and energy weighting detectors: a simulation study Phys. Med. Biol. 2005;50:5813 10.1088/0031-9155/50/24/004 16333157

[19] Gullikson EM. X-ray data booklet. Lawrence Berkeley National Laboratory, Berkeley, CA 2001; p. 1–38.

[20] Pemler P, Schneider U, Besserer J. Evaluation des Elektronendichte-Phantoms CIRS Model 62. Z Med Phys. 2001;11:25–32. 10.1016/S0939-3889(15)70384-3 11487856

[21] Hernandez AM, Boone JM. Tungsten anode spectral model using interpolating cubic splines: Unfiltered x-ray spectra from 20 kV to 640 kV. Med Phys. 2014;41:042101 10.1118/1.4866216 24694149 PMC3985923

[22] Lamperti PJ, O’Brien M. NIST Measurement Services: Calibration of x-ray and gamma-ray measuring instruments. NIST Special Publication. 2013;250-58:6.

[23] Mayo SC, Miller PR, Wilkins SW, Davis TJ, Gao D, Gureyev TE, et al Quantitative x-ray projection microscopy: Phase contrast and multi-spectral imaging. J Micro. 2002;207:79–96. 10.1046/j.1365-2818.2002.01046.x 12180954

[24] Experimental test of the intrinsic dimensionality of Hounsfield unit measurements 10.17504/protcols.iosw3efgn

[25] Raftery AE. Bayesian model selection in social research. Sociol Method. 1995;25:111–163.

[26] Weaver JE, Huddleston AL. Attenuation coefficients of body tissues using principal-components analysis. Med Phys. 1985;12:40–45. 10.1118/1.595759 3883118

[27] Han D, Porras-Chaverri MA, O’Sullivan JA, Politte DG, Williamson JF. Technical Note: On the accuracy of parametric two-parameter photon cross section models in dual-energy CT applications. Med Phys. 2017;44:2438–2446. 10.1002/mp.12220 28295418 PMC5473361

[28] McCollough CH, Bruesewitz MR, McNitt-Gray MF, Bush K, Ruckdeschel T, Payne JT, et al The phantom portion of the American College of Radiology (ACR) Computed Tomography (CT) accreditation program: Practical tips, artifact examples, and pitfalls to avoid. Med Phys. 2004;31:2423–2442. 10.1118/1.1769632 15487722

[29] Gammex. Tissue mimicking materials nominal characteristics. updated 2016-Mar-10. 2016;.

[30] Cropp RJ, Seslija P, Tso D, Thakur Y. Scanner and kVp dependence of measured CT numbers in the ACR CT phantom. J Appl Clin Med Phys. 2013;14:338–349. 10.1120/jacmp.v14i6.4417 PMC5714621 24257284

[31] Sande EPS, Martinsen ACT, Hole EO, Olerud HM. Interphantom and interscanner variations for Hounsfield units—establishment of reference values for HU in a commercial QA phantom. Phys Med Biol. 2010;55:5123–5135. 10.1088/0031-9155/55/17/015 20714048

[32] Garcia LIR, Azorin JFP, Almansa JF. A new method to measure electron density and atomic number using dual-energy CT images. Phys Med Biol. 2016;61:265–279. 10.1088/0031-9155/61/1/265 26649484

[33] Cai C, Legoupil S, Mohammad-Djaferi A. A full-spectral Bayesian reconstruction approach based on the material decomposition model applied in dual-energy computed tomography. Med Phys. 2013;40:111916 10.1118/1.4820478 24320449

[34] Taguchi K and Iwanczyk JS Vision 20/20: Single photon counting x-ray detectors in medical imaging Med. Phys. 2013;40:100901 10.1118/1.4820371 24089889 PMC3786515

[35] De Man B, Nuyts J, Dupont P, Marchal G, and Suetens P An iterative maximum-likelihood polychromatic algorithm for CT IEEE Trans. on Med. Imaging 2001;20:999–1008. 10.1109/42.959297 11686446

[36] Willemink MJ, de Jong PA, Leiner T, de Heer LM, Nievelstein RAJ, Budde RPJ, and Schilham AMR Iterative reconstruction techniques for computed tomography Part 1: Technical principles Eur. Radiol. 2013;23:1623–1631. 10.1007/s00330-012-2765-y 23314600

[37] Chantler CT. Theoretical form factor, attenuation, and scattering tabulation for *Z* = 1–92 from *E* = 10 eV to E = 0.4-1.0 MeV. J Phys Chem Ref Data. 1995;24:71–643. 10.1063/1.555974

[38] Rehr JJ. Theoretical approaches to x-ray absorption fine structure. Rev Mod Phys. 2000;72:621–654. 10.1103/RevModPhys.72.621

[39] Luo XF, Xie XQ, Cheng S, Yang Y, Yan J, Zhang H, et al Dual-energy CT for patients suspected of having liver iron overload: Can virtual iron content imaging accurately quantify liver iron content? Radiol. 2015;277:141856 10.1148/radiol.2015141856 25880263

[40] Hyodo T, Hori M, Lamb P, Sasaki K, Wakayama T, Chiba Y, et al Multimaterial decomposition algorithm for the quantification of liver fat content by using fast-kilovolt-peak switching dual-energy CT: Experimental validation. Radiol. 2017;282:160129 10.1148/radiol.2016160129 27541687

[41] Gutjahr R, Halaweish AF, Yu ZC, Leng S, Lu YF, Li ZBC, Jorgensen SM, Ritman EL, Kappler S. and McCollough CH, Human imaging With photon counting-based computed tomography at clinical dose levels: Contrast-to-noise ratio and cadaver studies Investigative Radiol. 2016;51:421–429 10.1097/RLI.0000000000000251 PMC4899181 26818529

